# Quantitative serological antibody testing for suspected neuroborreliosis

**DOI:** 10.1007/s00415-020-09721-7

**Published:** 2020-02-01

**Authors:** Christian Schneider, Jörg Gielen, Philip Röth, Philipp Albrecht, Michael Schroeter, Gereon R. Fink, Gilbert Wunderlich, Helmar C. Lehmann

**Affiliations:** 1 Department of Neurology, Faculty of Medicine and University Hospital Cologne, University of Cologne, Kerpener Straße 62, 50937 Cologne, Germany; 2 Institute for Medical Microbiology, Immunology and Hygiene (IMMIH), University of Cologne, Cologne, Germany; 3 Department of Neurology, Faculty of Medicine and University Hospital Düsseldorf, University of Düsseldorf, Düsseldorf, Germany; 4 Jülich Research Center, Institute of Neuroscience and Medicine (INM-3), Jülich, Germany; 5 Faculty of Medicine and University Hospital Cologne, Center for Rare Diseases, University of Cologne, Cologne, Germany

**Keywords:** Neuroborreliosis, Lyme disease, ELISA, CLIA

## Abstract

**Objective:**

To assess the importance of serum IgG/IgM antibody titers for the differentiation of Lyme neuroborreliosis (LNB) from its mimics.

**Method:**

This was a retrospective, cross-sectional study conducted at two German neurological centers. Serological parameters (ELISA or CLIA analysis) and clinical presentation of 28 patients with definite LNB were compared to those of 36 patients with neurological symptoms mimicking LNB (mimics). Analysis was performed using receiver operating characteristic (ROC) and binary logistic regression.

**Results:**

Elevated IgG-titers had a high sensitivity for neuroborreliosis in both centers (0.95 and 1.0). The optimal cutoff-values were set to 26.35 in center A (ELISA), and 64.0 in center B (CLIA). Diagnostic specificity was 0.41 and 0.89 in this constellation. Elevated IgM-titers showed a high diagnostic specificity for a cutoff at 68.10 (A) and 47.95 (B) (0.93 and 0.89). Sensitivity was 0.45 and 0.5. Overall diagnostic accuracy was low in both centers (A: IgG AUC = 0.665, IgM AUC = 0.629; B: IgG AUC = 0.917, IgM AUC = 0.556). In logistic regression of antibody titers and clinical measures, prediction of LNB was significantly better than the “null hypothesis”. Clinical measures showed the highest odds ratio.

**Conclusion:**

Data show that in addition to the clinical presentation of patients with symptoms suggesting central or peripheral nervous system manifestation, serum IgG- and IgM-titers help to identify LNB-patients. The results should guide physicians counseling patients with suspected LNB about further diagnostic steps and treatment.

## Introduction

Lyme borreliosis is an infectious disease transmitted by spirochetes of the six species in the spirochete family Borreliaceae. Its neurological manifestations are often referred to as Lyme neuroborreliosis (LNB). To establish the diagnosis, investigations of the cerebral spinal fluid (CSF) are recommended. International consensus demands a clinical sign suggestive of borreliosis (possible LNB); additional signs of inflammation in the CSF (pleocytosis, signs of blood–brain-barrier damage, intrathecal IgG synthesis) make LNB probable; and a pathological Borrelia-specific antibody index ascertains the diagnosis of LNB [1].

However, this diagnostic algorithm is difficult to apply in cases in which CSF examination is delayed or not feasible, e.g. in patients with anticoagulation.

Lumbar puncture itself is a safe procedure, although it can cause side effects such as headaches and even severe complications have rarely been reported [2, 3]. Nevertheless, the existence of asymptomatic seropositivity and the diverse clinical picture often cause uncertainty about diagnostic and treatment approaches [4-6].

Immunosorbent assays (ELISA) and chemiluminescence immunoassays (CLIA) used for routine serological testing provide quantification of antibody titers. We reasoned that LNB is typically associated with high serum levels for anti-Borrelia antibodies. Thus, we assessed the utility of serum antibody titers as a complementary approach to discriminate LNB-patients from its mimics in a preselected cohort of patients with symptoms suggesting central or peripheral nervous system manifestation.

## Material and methods

### Patients

Twenty-eight patients with confirmed LNB according to the guidelines of the German Neurological Society were included (see Table 1). Patients were clinically evaluated and diagnosed either at the Department of Neurology at the University Hospital of Cologne (center A) or Düsseldorf (center B). Moreover, 36 patients (see Table 1) with suspected neuroborreliosis, later referred to as LNB-mimics, were included (both centers). Patients with suspected LNB presented with clinical signs suggesting central or peripheral nervous system involvement and elevated antibody titers (either IgG, IgM, or both) against Borrelia burgdorferi in peripheral blood samples. All patients received a complete diagnostic work-up regarding nervous system manifestation of borreliosis, including CSF investigations on cell count, lactate, glucose, protein, specific antibody titers (IgG, IgM) against Borrelia burgdorferi, and specific antibody index. For LNB-mimics, CSF investigation excluded neuroborreliosis (no pleocytosis, no pathological Borrelia-specific antibody index).

**Table 1:** Clinical characteristics of patients (both centers)

Characteristics	Lyme Neuroborreliosis	Mimics
Age (mean, SD)	43 ± 17	45 ± 15
Gender (female/male)		
Female	22/28	21/36
Male	6/28	15/36
Tick bite	8/28	8/36
Erythema migrans previously	5/28	6/36
Duration of symptoms		
0–1 month	21	17
> 1 month	7	19
Symptoms		
Cranial neuropathy	15/28	4/36
Radiculopathy	9/28	4/36
Meningopathy	5/28	7/36
Fatigue	1/28	6/36
Others	4/28	21/36
Previous LNB	1/28	1/36
Cell count/µl CSF (mean, SD)	207 ± 145	< 5

Patients presenting with concomitant cutaneous or other manifestations (e.g., arthritis) were not included.

### Borrelia Immunosorbent assays (ELISA)

All samples (peripheral blood samples, cerebral spinal fluid) of center A were analyzed by the MIKROGEN recomWell Borrelia IgG- and IgM-specific ELISA. The ELISA uses recombinant Borrelia burgdorferi antigens (IgM: OspC, p41, VlsE; IgG: p100, OspC, VlsE, p18). The analysis was performed following the manufacturer’s instructions. Briefly, all samples were incubated with specific antigens for one hour at 37 °C. After a series of four washes, samples were incubated with diluted horseradish peroxidase-conjugated anti-human IgG or IgM. After another four washes, samples were developed with chromogenic substrate tetramethylbenzidine. The reaction was stopped using 24.9% phosphoric acid, and the optical density was afterwards measured spectrophotometrically at 450 and 650 nm. Extinction values were assigned to antibody activity in U/ml according to the manufacturer’s protocol. Values > 24 U/ml were deemed positive.

### Borrelia chemiluminescence assay (CLIA)

At center B, all samples were analyzed using the Liaison Borrelia burgdorferi chemiluminescent immunoassay by DiaSorin. Briefly, samples were incubated with paramagnetic particles coated with recombinant OspC (IgM) or VlsE (IgG) antigens and with a tracer labeled with an isoluminol derivate. After removing unbound material by a wash cycle, a starter reagent was added, and a chemiluminescence reaction was induced. The light signal was then measured by a photomultiplier as relative light units and later converted to U/ml. Values > 22 U/ml (IgM) and > 15 (IgG) in blood samples and > 3.5 (IgM) and > 5.5 (IgG) in CSF were deemed positive.

All positive ELISA or CLIA results were confirmed by Western blot.

### Statistical analysis

Sensitivity and specificity of quantitative IgG and IgM-titers were evaluated by receiver operating characteristic (ROC) analysis. Accuracy of the tests was determined by area under the curve (AUC)- values. Binary logistic regression was used to predict neuroborreliosis from IgG, IgM-titers, and clinical measures. For this purpose, clinical characteristics were divided into a “neuroborreliosis cluster” and an “unspecific cluster”. The “neuroborreliosis cluster” included radiculitis, cranial neuropathy, and meningopathy, other symptoms were assigned to the “unspecific cluster”. ELISA and CLIA-titers of neuroborreliosis patients were compared to those of disease mimics by Mann–Whitney *U* Test. A *p*-value < 0.05 was considered statistically significant. Statistical analyses were performed using IBM SPSS Statistics (Version 25.0. Armonk, NY: IBM Corp.).

## Results

Patients’ symptoms and signs on admission are summarized in Table 1. All patients presented with symptoms suggesting nervous system manifestation of borreliosis. In confirmed cases of LNB, cranial nerve involvement (53.6% of all LNB-patients versus 11.1% of mimics) and radicular involvement (32.1% of all LNB-patients versus 11.1% of mimics) occurred frequently, whereas most LNB-mimics presented with complaints such as fatigue or paresthesia (75% of all mimics versus 17.9% of LNB). Subsequently, headaches suspected to be a consequence of an infectious involvement of the meninges are referred to as meningopathy. The frequency of meningopathy was similar in the two groups (17.9% of LNB-patients versus 19.4% of mimics).

Quantitative evaluation of IgG- and IgM-titers against Borrelia burgdorferi in peripheral blood samples of LNB-patients and mimics, as well as IgG- and IgM-titers in CSF of patients with LNB, are presented in Table 2. In both centers (A, B), the mean IgG- and IgM-titers in peripheral blood samples of patients with LNB were higher compared to those in LNB-mimics. However, these results showed no statistical significance due to high standard deviations (SD). Elevated serum and CSF-titers were observed in both centers. Yet, as the applied assays were not the same in both centers, the extent of serum and CSF-titers were quantitatively different.

**Table 2:** Quantitative evaluation (mean, SD) of immunosorbent assays (center A) and chemiluminescence immunoassays (center B) against Borrelia burgdorferi in serum and cerebrospinal fluid (CSF) of patients (LNB, Lyme Neuroborreliosis and mimics) of two medical centers (A, B)

LNB	IgG Serum	IgM Serum	Mimic	IgG Serum	IgM Serum
Center A (*n* = 20)	859.7 ± 1626	444.4 ± 1106	Center A (*n* = 27)	284.0 ± 596.2	39.4 ± 51.9
Center B (*n* = 8)	190.0 ± 57.2	55.3 ± 54.7	Center B (*n* = 9)	35.1 ± 77.0	34.0 ± 22.0

	IgG CSF	IgM CSF
Center A	1803 ± 2627.9	211.9 ± 299.4
Center B	174.6 ± 95.1	98.3 ± 87.8

**Fig. 1:**
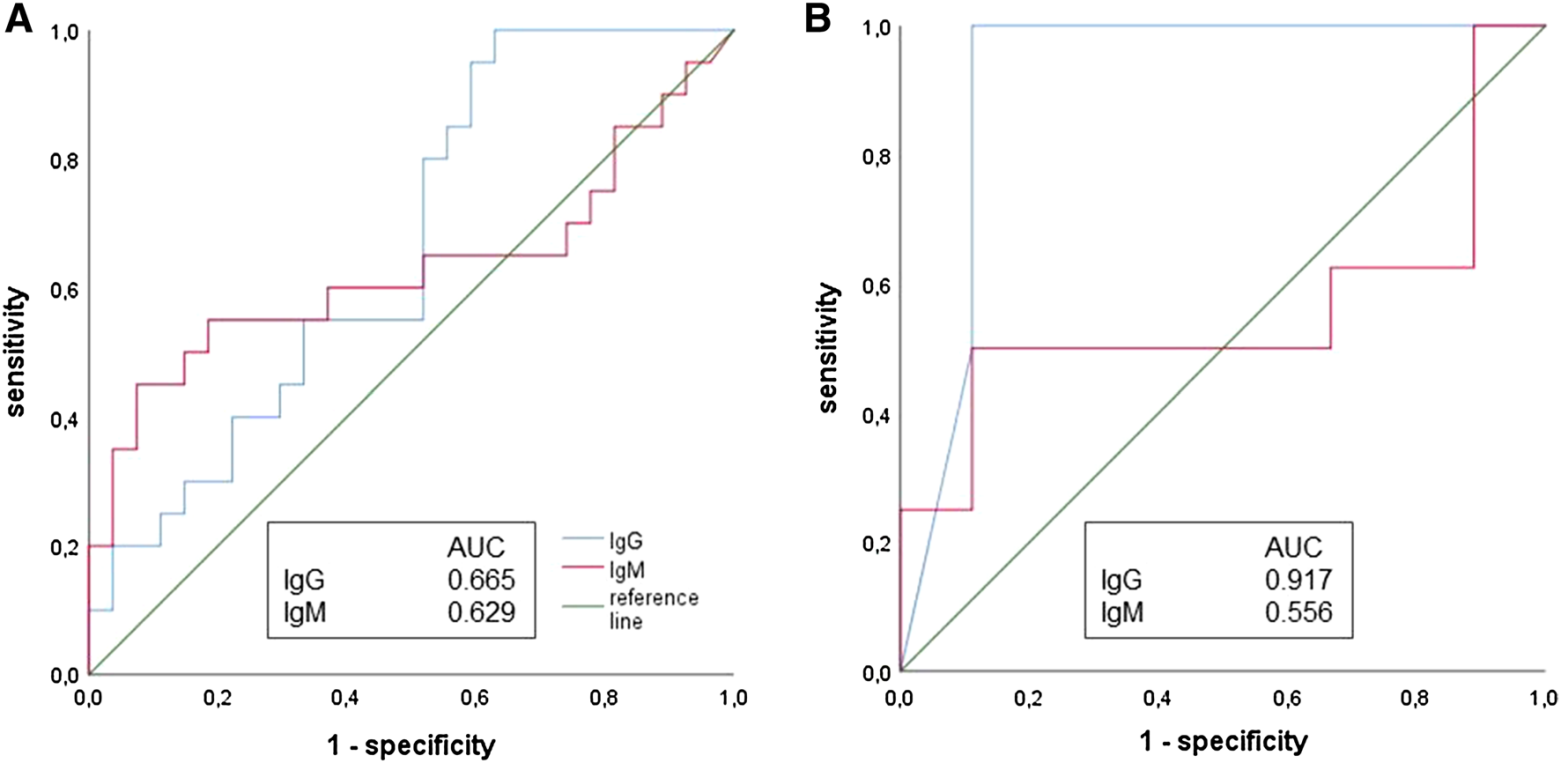
Receiver operating characteristic (ROC)-analysis of IgG and IgM-titers in center A (**a**) and center B (**b**). Diagnostic accuracy was evaluated by the area under the curve (AUC)

To assess the utility of the serum IgG and IgM antibody titer as complementary diagnostic biomarkers for LNB, we performed ROC-analysis (Fig. 1). Elevated IgG-titers had a high sensitivity for neuroborreliosis in both centers (0.95 and 1.0). The optimal cutoff-values were set to 26.35 in center A (ELISA), and 64.0 in center B (CLIA). Diagnostic specificity was 0.41 and 0.89 for these cutoff-values. Elevated IgM-titers showed a high diagnostic specificity for a cutoff at 68.10 (A) and 47.95 (B) (0.93 and 0.89). Sensitivity was 0.45 and 0.5 in this constellation. Overall diagnostic accuracy was low in both centers, except IgG AUC-analysis in center B showed good performance (A: IgG AUC = 0.665, IgM AUC = 0.629; B: IgG AUC = 0.917, IgM AUC = 0.556).

Additionally, in center A we performed logistic regression to predict LNB from IgG and IgM-titers, as well as IgG, IgM-titers and clinical measures (Table 3). Both models were superior to the “null model” to predict LNB according to “Omnibus Tests of Model Coefficients”. However, when including clinical measures p-values were considerably smaller (*p*-value 0.016 versus < 0.001). When IgG and IgM-titers were considered, 68.1% of the cases were classified correctly compared to 57.4% in the baseline model. In contrast, when also including clinical measures 85.1% were classified correctly. In analysis of “the variables in the equation”, clinical measures presented with a high odds ratio (61.794), whereas the odds ratios of IgG and IgM were low (1.001 and 1.009).

**Table 3:** Binary logistic regression of IgG, IgM-titers and clinical measures of center A to predict neuroborreliosis

IgG/IgM/Clinical measures		*χ*^2^	*df*	Sig.	IgG/IgM	*χ*^2^	*df*	Sig
Step 1	Step	32,322	3	0,000	Step	8,327	2	0,016
	Block	32,322	3	0,000	Block	8,327	2	0,016
	Model	32,322	3	0,000	Model	8,327	2	0,016

IgG/IgM/Clinical measures		B	S.E.	Wald	*df*	Sig	Exp(B)
Step 1	IgG	0,001	0,001	3,487	1	0,062	1,001
	IgM	0,009	0,008	1,123	1	0,289	1,009
	Clinical	4,124	1,179	12,239	1	0,000	61,794
	Constant	− 3,698	1,256	8,665	1	0,003	0,025

Upper table shows “Omnibus Test of Model Coefficients”. On the left upper side tests for IgG, IgM and clinical measures as explanatory variables are displayed. On the right upper side tests for IgG, IgM without clinical measures are displayed. Both models are significantly better than the “null model” in predicting neuroborreliosis. On the bottom table the “Variables in the Equation” are displayed which show high odds ratio for clinical measures

*df *degree of freedom,* Sig. *significance,* B *coefficient,* S.E. *standard error,* Wald *Wald **χ**^**2**^ value,* Exp*(*B*) odds ratio

## Discussion

Findings of our study show that in patients with symptoms suggestive for LNB, serum IgG-titers may help to exclude true LNB-cases. Moreover, elevated IgM-titers can help to distinguish LNB-patients from disease mimics. However, overall the diagnostic accuracy of serum titers for discrimination of neuroborreliosis was low, demonstrated by low AUC-values.

Additional analyses by logistic regression showed that serum-titers help to improve prediction of LNB over the “null model”, however the significance of these variables was very low. In contrast, clinical measures that are associated with the disease in its classical form such as radiculitis helped to predict LNB best which was reflected by a high odds ratio.

Our data show the potential and limitations of quantitative serum titers in this preselected cohort. Despite the overall rather poor performance of antibody titers, publications on other surrogate markers such as d-dimers for diagnosis of thrombotic events  underline the importance of these measures, especially to exclude a disease and to refrain from further diagnostic steps [7, 8].

Recent data support the potential of quantitative ELISA assessment in LNB, and Zwerink and colleagues reasoned that in highly positive ELISA titer constellations, even immunoblot may be omitted [9]. Even though in our cohort elevated IgG-titers showed a high sensitivity (0.95 and 1.0), LNB can rarely also occur with normal IgG/IgM serum titers, especially in patients with a short history of symptoms [1, 6]. There were no definite LNB-patients with normal IgG-titers in our cohort. However, no PCR-studies of CSF were performed to detect Borrelia species, which could have made the diagnosis definite in case of a negative antibody result.

Our data also demonstrate noticeable differences of serum titers between ELISA and the CLIA assays used. These findings are in line with other studies on ELISA and CLIA assays showing that these assays have discrepancies in quantitative measurement but sound overall analytical agreement [10-12]. Moreover, recent data also suggest that even titers of different quantitative ELISA assays cannot be compared without further consideration [13, 14].

Overall our study shows the potential of both ELISA and CLIA to exclude LNB-cases and distinguish patients from disease mimcs. Nevertheless, especially the diagnostic accuracy of IgG-titers differed in both centers. As we did not perform both tests in the same patient, and the overall number of patients investigated by CLIA was low, the results of center B should be interpreted with caution.

Another limiting factor of our study is that none of the patients suffered from concomitant cutaneous affection or arthritis. Thus, our results are only applicable to a cohort with symptoms suggestive for nervous system affection and elevated serum titers. Whether or not the clinical utility of the identified parameters extends to patients with coexisting manifestations of borreliosis remains to be investigated.

Moreover, one should keep in mind that serological titers are neither suitable to verify the success of treatment nor differentiate active disease from the persistence of antibodies nor re-infection. Treatment may occur accidentally since antibiotic treatment with cephalosporins, tetracycline, or penicillin derivates may be initiated for multiple other causes but may efficiently treat LNB at the same time. To differentiate these putative confounds, CSF analysis is indispensable.

In conclusion, these data show that quantitative serological antibody testing can help to differentiate LNB from its mimics. In the case of a patient with symptoms suggesting central or peripheral nervous system affection, 2.8 fold (or more) elevated IgM-titers should lead to immediate initiation of therapy when a lumbar puncture is not feasible or contraindicated. Furthermore, these data help to refrain from further or even more invasive diagnostic investigations, especially when IgG serum titers are normal and a longer history of symptoms is present.
